# Synthesis of Pd-Doped SnO_2_ and Flower-like Hierarchical Structures for Efficient and Rapid Detection of Ethanolamine

**DOI:** 10.3390/molecules29153650

**Published:** 2024-08-01

**Authors:** Wenjie Bi, Jinmiao Zhu, Bin Zheng, Shantang Liu, Lilong Zhang

**Affiliations:** 1 School of Chemistry and Pharmaceutical Engineering, Hefei Normal University, Hefei 230601, China; 2 Institute of Solid-State Physics, Hefei Institutes of Physical Science, Chinese Academy of Sciences, Hefei 230031, China; 3 Key Laboratory for Green Chemical Process of Ministry of Education, School of Chemistry and Environmental Engineering, Wuhan Institute of Technology, Wuhan 430074, China; 4 National Key Laboratory of Green Pesticide, Key Laboratory of Green Pesticide and Agricultural Bioengineering, Ministry of Education, State-Local Joint Laboratory for Comprehensive Utilization of Biomass, Center for R&D of Fine Chemicals of Guizhou University, Guizhou University, Guiyang 550025, China

**Keywords:** Pd-SnO_2_ hierarchical structure, gas sensor, ethanolamine detection

## Abstract

In this study, we successfully synthesized a Pd-doped SnO_2_ (Pd-SnO_2_) material with a flower-like hierarchical structure using the solvothermal method. The material’s structural proper-ties were characterized employing techniques such as XRD, XPS, FESEM and HRTEM. A gas sensor fabricated from the 2.0 mol% Pd-SnO_2_ material demonstrated exceptional sensitivity (R_a_/R_g_ = 106) to 100 ppm ethanolamine at an operating temperature of 150 °C, with rapid response/recovery times of 10 s and 12 s, respectively, along with excellent linearity, selectivity, and stability, and a detection limit down to 1 ppm. The superior gas-sensing performance is attributed to the distinctive flower-like hierarchical architecture of the Pd-SnO_2_ and the lattice distortions introduced by Pd doping, which substantially boost the material’s sensing characteristics. Further analysis using density functional theory (DFT) has revealed that within the Pd-SnO_2_ system, Sn exhibits strong affinities for O and N, leading to high adsorption energies for ethanolamine, thus enhancing the system’s selectivity and sensitivity to ethanolamine gas. This research introduces a novel approach for the efficient and rapid detection of ethanolamine gas.

## 1. Introduction

In recent years, with the development of technology, environmental pollution has gradually attracted widespread attention. Some pollutants can cause severe irritation to the respiratory tract, or even damage to the nervous system and brain [1,2]. One such pollutant is ethanolamine (EA), a typical volatile organic compound (VOC) that is widely used in laboratories and manufacturing industries. It has the characteristics of high stability and low degradability. Researchers have found that EA can cause irritation to the skin and eyes, and prolonged exposure can even cause damage to the nervous system [3,4]. Therefore, it is of paramount importance to conduct real-time monitoring of EA content in the air.

Metal oxides (MOs) such as ZnO [4], SnO_2_ [5], In_2_O_3_ [6], and WO_3_ [7] are widely used for the detection of toxic and harmful gases due to their good stability and response to VOCs [8]. Because of its excellent physical and chemical properties, and its electrical performance, SnO_2_ has attracted much attention in the field of gas detection [9,10]. According to reports in the literature, the microscopic morphologies of metal oxide gas-sensitive materials are closely related to their gas-sensing properties [11]. Differences in micromorphology can affect parameters such as the specific surface area of a material, leading in turn to changes in its gas-sensing properties. Compared with other micro-morphologies, three-dimensional hierarchical structures exhibit excellent stability and are less prone to aggregation. These unique structures can also provide a larger surface area, promoting the rapid diffusion of gases [12]. For instance, using a one-step hydrothermal method, Zhu et al. prepared a Y-doped SnO_2_ hierarchical structure and used it for formaldehyde detection. Its excellent gas-sensing performance was attributed to Y doping and a unique 3D hierarchical structure [13]. Similarly, using a hydrothermal method, Hu et al. prepared a NiO/SnO_2_ hierarchical structure and used it for acetone detection. Their results showed that the NiO/SnO_2_ hierarchical structure exhibited excellent sensing performance for acetone [14]. Studies like these have shown that preparing a unique three-dimensional hierarchical structure can effectively enhance the gas-sensing performance for VOCs.

For pure metal oxides, in addition to enhancing gas-sensing performance by changing morphologies, metal-ion doping can also be used to optimize gas-sensing performance. Doping metal ions can cause lattice distortion and increase active sites on the surface of materials, as has been widely reported [15,16]. Shang et al. prepared Fe-doped NiO using a one-step hydrothermal method and found that it exhibited excellent gas-sensing performance for butanone; this was attributed to changes in the carrier concentration and an increase in oxygen vacancies caused by Fe doping [17]. Li et al. prepared Cu-doped SnO_2_ using a simple hydrothermal method and then used it to detect trace amounts of SO_2_. Its excellent gas-sensing performance was attributed to enhanced chemical activity on the surface of the material due to Cu doping [18]. As a precious metal, Pd possesses a unique electronic structure and excellent catalytic properties, which substantially amplify the gas-sensing capabilities of semiconductor materials. Compared to other transition metals, Pd exhibits superior thermal stability, allowing it to maintain superior performance even at the elevated operational temperatures of sensors. Through Pd doping, the bandgap and Fermi level of SnO_2_ can be adjusted, thereby improving its electronic characteristics. This optimization of the electronic structure helps to increase the sensitivity to target gases, which is crucial for enhancing the detection performance of sensors. Moreover, Pd doping enriches the active sites on the SnO_2_ surface, which are pivotal for the effective chemical adsorption of gas molecules [19,20,21]. In summary, doping with Pd is now considered an effective method to further enhance the gas-sensing properties of materials. However, there have been few reports on the preparation of Pd-SnO_2_-sensitive materials with flower-like hierarchical structures for use in studies of sensitivity to ethanolamine gas.

In the present study, we successfully synthesized Pd-SnO_2_ flower-like hierarchical structures with different doping ratios using a one-step hydrothermal method. The sensor based on Pd-SnO_2_ was found to exhibit an excellent gas-sensing performance in detecting ethanolamine at an optimal operating temperature, as well as superb stability and selectivity. We also analyzed the gas-sensing mechanism of the prepared Pd-SnO_2_ sensitive material using first principles. In short, we developed an effective method for preparing Pd-SnO_2_ with a flower-like hierarchical structure which delivered improved detection performance for ethanolamine in ambient air.

## 2. Results and Discussion

**Figure 1:**
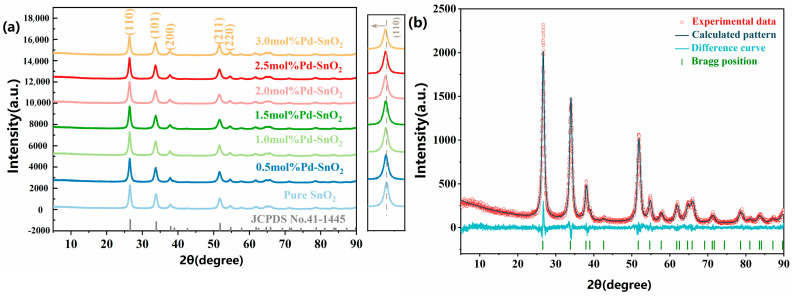
(**a**) XRD patterns of samples with different Pd doping ratios, (**b**) the typical Rietveld refinement data.

The crystal structure of the samples was analyzed via XRD, as shown in Figure 1a. As can be observed from the XRD patterns, the diffraction peaks of all samples corresponded with the characteristic peaks of SnO_2_ in the tetragonal rutile structure (JCPDS File No. 41-1445) [22]. Moreover, no diffraction peaks of any other impurities were detected in the spectrum, indicating that the synthesized samples possessed a high degree of purity. The observation of strong diffraction peak intensities and narrower full widths at half-maxima (FWHM) allows us to indicate that the synthesized samples exhibited good crystallinity. Previous studies have suggested that the close similarity in ionic radii between Pd^2+^ (0.86 Å) and Sn^4+^ (0.71 Å) facilitates the substitution of Pd^2+^ for Sn^4+^, allowing for the integration of Pd^2+^ into the crystal lattice structure of SnO_2_ [1,23]. Compared with those of pure SnO_2_, the XRD patterns of samples doped with Pd show shifts in their diffraction peaks towards lower angles. This phenomenon can be explained by the Bragg equation, which can be expressed as n λ = 2d sin θ, where n and λ are constants, d is the lattice spacing, and θ is the Bragg diffraction angle. In this equation, the Bragg diffraction angle (θ) and the lattice spacing (d) are the two key parameters [24]. It can be inferred that when the value of d increases, this leads to a decrease in θ. In this study, we doped Pd^2+^ into the lattice of SnO_2_, and this resulted in an increase in the lattice spacing (the values for pure SnO_2_ and 2 mol%Pd-SnO_2_ are 0.3451 nm and 0.3465 nm, respectively). Consequently, there was a slight decrease in the corresponding diffraction angle. As the concentration of Pd^2+^ increased, the crystallinity of the sample gradually decreased, and the corresponding diffraction peaks broadened. This phenomenon indicates that doping with Pd^2+^ effectively inhibits the growth of SnO_2_ crystal grains.

The XRD patterns of the synthesized Pure SnO_2_ samples underwent Rietveld full-spectrum fitting and structural refinement using TOPAS software Version 6. Figure 1b displays the refinement results, with the specific refined parameters detailed in Table 1. The sample’s average grain size is determined to be approximately 9.3810 nm. The refined lattice parameters are a = b = 4.7427 Å and c = 3.1907 Å. It is evident from Figure 1b that the line broadening, attributed to the small crystal size and microstrain brought in by the heterosubstitution, influenced the final agreement between the experimental and calculated patterns. Nonetheless, the difference curve confirms that the correspondence between the calculated and experimental XRD patterns is satisfactory [25,26,27].

**Figure 2:**
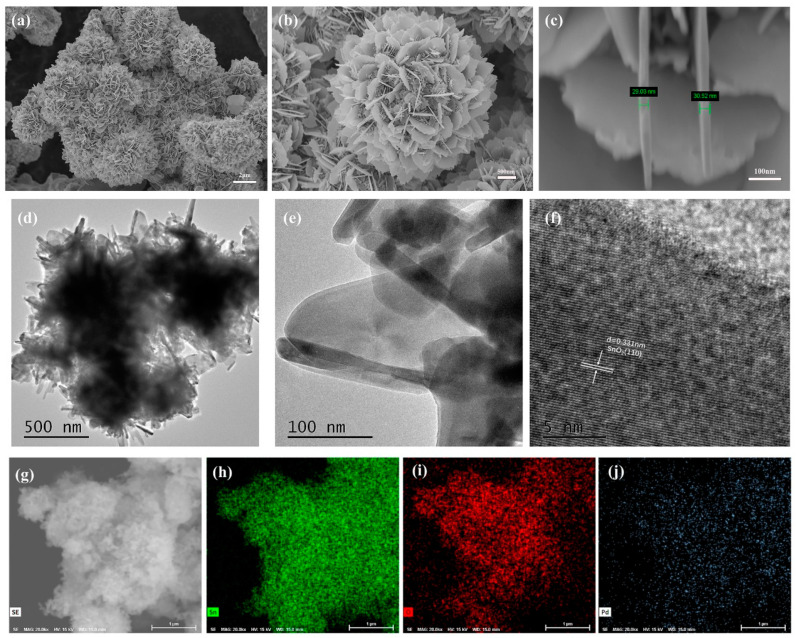
(**a**–**c**) FEESM, (**d**,**e**) TEM, (**f**) HRTEM, (**g**–**j**) FESEM-EDX image of 2.0 mol% Pd-SnO_2_.

We characterized the morphology and composition of the samples using field emission scanning electron microscopy (FESEM). Figure 2a–c presents FESEM images of the 2.0 mol% Pd-SnO_2_ sample at different magnifications. As can be seen from Figure 2a,b, the prepared samples consisted of intersecting nanosheets stacked on top of each other, resulting in a flower-like hierarchical structure. Almost no other morphologies were present, indicating that the samples had excellent morphological uniformity. From high-magnification SEM images, it was also observed that these nanosheets had uniform and smooth surfaces, with neat and clearly discernible edges. Further measurements indicated that the thickness of these nanosheets was approximately 30 nm, as can be seen in Figure 2c. As reported in the literature, altering the doping ratio in SnO_2_ is an effective strategy for modulating the surface roughness and controlling the morphology of the material. Initially, varying doping ratios can markedly transform the morphology of materials, exemplified by transitions from spherical to rod-like shapes or from flake-like to fibrous forms. These morphological alterations significantly influence the specific surface area and pore structure, which in turn affect the gas sensing capabilities of the materials. Subsequently, the doping process augments surface roughness, and this rough surface provides more active sites, enhancing the sensitivity of the sensor. The above phenomena and effects have been widely confirmed in previous studies [14,28].

We further confirmed the composition and microstructure of samples using transmission electron microscopy (TEM) and high-resolution transmission electron microscopy (HRTEM). In Figure 2d,e, which shows TEM images of a 2.0 mol% Pd-SnO_2_ sample, we can clearly observe a flower-like hierarchical structure composed of nanosheets. The shape and size of this structure are essentially consistent with the observations made via FESEM. Figure 2f presents a HRTEM image of 2.0 mol% Pd-SnO_2_. The measured lattice spacing is 0.331 nm, which corresponds to the (110) crystal plane of the tetragonal rutile structure of SnO_2_. Utilizing energy-dispersive X-ray spectroscopy (FESEM-EDX) techniques, we analyzed the elemental distribution of a 2.0 mol% Pd-SnO_2_ sample. The results are presented in Figure 2g–j. Through elemental mapping analysis, we found that Sn, O, and Pd elements exhibit a uniform distribution within the flower-like hierarchical structure of Pd-SnO_2_.

**Figure 3:**
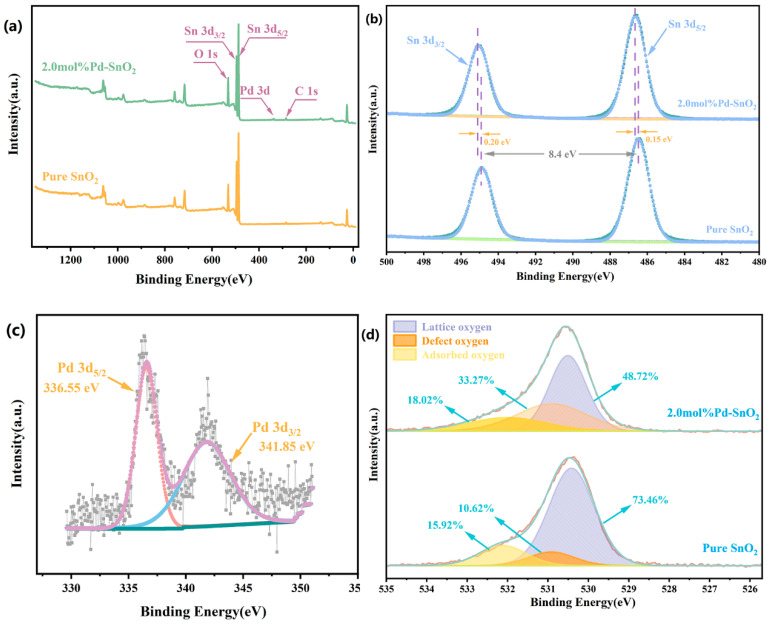
(**a**) XPS survey spectra, (**b**) Sn 3d, (**c**) Pd 3d, (**d**) O 1s in the pure SnO_2_ and 2 mol% Pd-SnO_2_.

X-ray photoelectron spectroscopy (XPS) was employed to characterize the composition and chemical valence states of pure SnO_2_ and 2.0 mol%Pd-SnO_2_ samples, and the results are presented in Figure 3. The calibration of all binding energy peaks was performed using the C1s peak, located at 284.6 eV, as a reference standard. Figure 3a illustrates the XPS full spectrum of pure SnO_2_ and 2 mol% Pd-doped SnO_2_ samples. The spectrum exhibits peaks exclusively associated with Sn, O, Pd, and C, indicating an absence of extraneous impurities. Figure 3b presents the high-resolution XPS spectra of the Sn 3d. Two distinct peaks can be observed at binding energies of approximately 494.9 eV and 486.5 eV, corresponding to the Sn 3d_3/2_ and Sn 3d_5/2_ spin–orbits, respectively, of Sn^4+^ ions in SnO_2_. The difference in binding energy between these peaks is 8.4 eV, which is consistent with previously reported findings [29,30]. In the 2.0 mol% Pd-SnO_2_ sample, compared with pure SnO_2_, the peaks of Sn 3d_3/2_ shifted towards higher binding energy by 0.20 eV, and the peaks of Sn 3d_5/2_ shifted towards higher binding energy by 0.15 eV. The shift in binding energy can be attributed to two primary factors. First, Pd, being a transition metal with a strong electron affinity, may attract electrons from Sn^4+^ when doped into its lattice. This electron transfer results in a higher binding energy for the Sn 3d [31,32]. Second, the difference in atomic radius between Pd and Sn can induce lattice distortions upon doping, potentially altering the electronic state of the Sn atoms. The high-resolution spectrum of Pd 3d in Figure 3c shows two distinct peaks at 336.55 eV and 341.85 eV. These peaks correspond to the Pd 3d_5/2_ and Pd 3d_3/2_ electronic states, respectively, indicating that palladium is present in the Pd-SnO_2_ sample in the Pd^2+^ oxidation state [33]. To investigate changes in the types and content of surface oxygen species before and after doping, we analyzed the O1s high-resolution spectra of pure SnO_2_ and 2 mol% Pd-SnO_2_ samples, as depicted in Figure 3d. The O1s peaks for all samples exhibited asymmetry and could be fitted with three Gaussian peaks. The peak at a binding energy of approximately 530.45 eV is attributed to lattice oxygen in SnO_2_ (O_L_). The peak at approximately 530.95 eV originates from surface defects of the material, such as oxygen vacancy (O_V_) defects. The peak at approximately 532.20 eV represents oxygen adsorbed on the surface of the material (O_C_) [34]. We measured the relative content percentages of three different oxygen species. In pure SnO_2_, the content levels of O_L_, O_V_, and O_C_ were 73.46%, 10.62%, and 15.92%, respectively. In contrast, in the 2 mol% Pd-SnO_2_ sample, the content levels of O_L_, O_V_, and O_C_ were 48.72%, 33.27%, and 18.02%, respectively. According to previous reports in the literature, the contents of O_V_ and O_C_ are closely related to gas-sensing performance [35,36]. In the 2 mol% Pd-SnO_2_ sample, the content ratios of O_V_ and O_C_ both increased. This suggests that the sample’s surface provided more active sites and exhibited enhanced oxygen adsorption capability, thereby enabling high-performance detection of target gases.

When Pd^2+^ is doped into the crystal lattice structure of SnO_2_, it is necessary to maintain charge balance by creating oxygen vacancies. This is required to keep the SnO_2_ crystal unit electrically neutral. The incorporation of a metal with a lower charge, while preserving the anionic sublattice, necessitates the formation of a significant accumulation of negative charge. Consequently, the formation of oxygen vacancies is necessary to ensure the electrical neutrality of the crystal unit [37,38].

## 3. Gas-Sensing Performance

**Figure 4:**
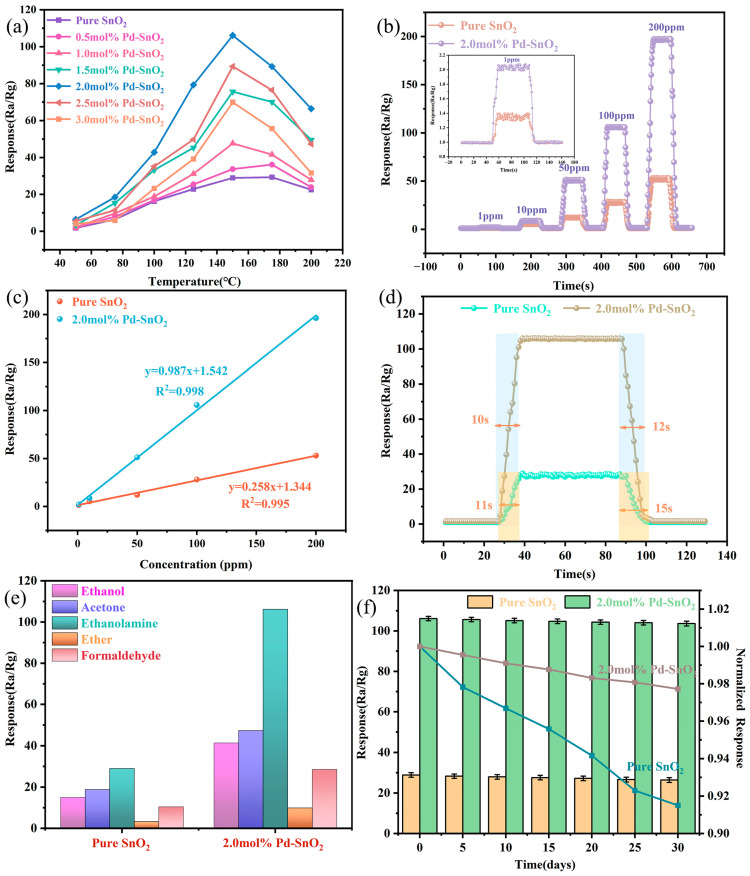
(**a**) Response curves of samples with different Pd-doping ratios to 100 ppm ethanolamine at different temperatures. (**b**) Response/recovery curves of pure SnO_2_ and 2 mol% Pd-SnO_2_ gas sensors to 1–200 ppm ethanolamine. (**c**) Fitting linear relationship between gas-sensing response and ethanolamine concentration. (**d**) Response/recovery curves for pure SnO_2_ and 2 mol% Pd-SnO_2_ gas sensors to 100 ppm ethanolamine. (**e**) Selectivity curves of pure SnO_2_ and 2 mol% Pd-SnO_2_ gas sensors to various gases at 150 °C. (**f**) Long-term stability of pure SnO_2_ and 2 mol% Pd-SnO_2_ gas sensors to 100 ppm ethanolamine at 150 °C.

As working temperature changes, the adsorption capacity of target gas molecules and the oxidation reaction rate on metal oxide semiconductor materials are both affected, and these changes subsequently impact the gas-sensing performance of the material. Identifying the optimal working temperature is therefore crucial for detecting target gases. Figure 4a displays response curves of gas sensors fabricated from Pd-SnO_2_ samples with various doping ratios (including 0 mol%, 0.5 mol%, 1.0 mol%, 1.5 mol%, 2.0 mol%, 2.5 mol%, and 3.0 mol%) to 100 ppm ethanolamine at different operating temperatures. From the graph, it is evident that within the temperature range of 50 °C to 200 °C all samples show a similar trend: their response values to ethanolamine initially increase and then decrease. It can also be seen that at an operating temperature of 150 °C, sensors fabricated from pure SnO_2_ and 2 mol% Pd-SnO_2_ exhibit optimal response values of 28.95 and 106.15, respectively. The response value of the 2 mol% Pd-SnO_2_ was approximately 3.67 times higher than that of pure SnO_2_, indicating that Pd doping significantly enhances the gas-sensing performance of SnO_2_.

Figure 4b shows the dynamic response curves of pure SnO_2_ and 2 mol% Pd-SnO_2_ gas sensors to 1, 10, 50, 100, and 200 ppm ethanolamine. These curves indicate that both sensors responded rapidly to various concentrations of ethanolamine and exhibited good recovery performance upon exposure to air. Both types of sensors exhibited excellent response/recovery characteristics upon observation. Further experiments revealed that the response value of the gas sensor increased with increasing ethanolamine concentration, although its growth rate gradually tended to plateau. This trend is likely associated with the active sites on the surface of the sensitive material approaching saturation [5]. The minimum concentration of ethanolamine that was measurable in this experiment was 1 ppm. The experimental results showed that these sensors exhibited response values of 1.3 and 1.9 to 1 ppm ethanolamine, as detailed in the inset of Figure 4b. The response values of the pure SnO_2_ and 2 mol% Pd-SnO_2_ gas sensors to various concentrations of ethanolamine were linearly fitted, and the results are shown in Figure 4c. Within the range of ethanolamine concentration from 1 ppm to 200 ppm, both sensors demonstrated a good linear relationship between the response values and gas concentration, with values of 0.995 and 0.998, respectively, for the correlation coefficient R^2^. 

It is well known that, in gas-sensing detection, response and recovery times are key parameters for measuring sensor performance. Figure 4d presents the response/recovery curves of two sensors exposed to 100 ppm ethanolamine. The response time and recovery time of the pure SnO_2_ gas sensor are 11 s and 15 s, respectively. The response time and recovery time of the 2 mol%Pd-SnO_2_ gas sensor are 10 s and 12 s, respectively. The reduction in response and recovery times may be attributed to the lattice defects introduced by Pd doping. These defects serve as additional adsorption sites, increasing the adsorption capacity for gas molecules and thereby accelerating the response and recovery processes.

Selectivity is a critical metric for assessing a sensor’s ability to differentiate between various types of gases. Good selectivity significantly enhances the practicality of sensors and the meeting of specific detection requirements. In the present study, to assess the selectivity of the materials, we compared the gas-sensing responses of sensors fabricated from pure SnO_2_ and 2 mol% Pd-SnO_2_ to 100 ppm concentrations of ethanol, acetone, ethanolamine, ether, and formaldehyde. The experimental results are presented in Figure 4e. Notably, the 2 mol% Pd-SnO_2_ gas sensor demonstrated a significantly higher response to ethanolamine, compared with other VOCs. The response intensity of this sensor to ethanolamine was more than double that of its response to other gases at the same concentration, indicating its excellent selectivity for ethanolamine.

From the perspective of practical applications, long-term stability is a critical indicator for assessing sensor performance. To assess the long-term stability of the gas sensor, a 30-day testing protocol was established. Throughout this period, the sensors’ response to 100 ppm ethanolamine was measured every 5 days, accompanied by a normalized analysis: specifically, the ratio of the sensors’ response on each subsequent day to that on the initial day. The test results are presented in Figure 4f. Upon examining the graph, it is evident that as time progresses, the normalized response curves for both samples exhibit a downward drift. This trend is likely due to the aging of the sensitive material or to environmental condition fluctuations. In comparison, the curve for 2.0 mol% Pd-SnO_2_ shows a less pronounced rate of decline compared to that of pure SnO_2_. This indicates that Pd doping significantly enhances the sensor’s stability and effectively slows the rate of performance degradation. Such enhancement is essential for ensuring the sensors’ consistent performance in practical applications.

Finally, we compared the gas-sensing performance of a 2.0 mol% Pd-SnO_2_ sensor for detecting ethanolamine with data obtained from the literature, as detailed in Table 2. The table shows that the sensor introduced in this article could maintain a high response value even at lower operating temperatures, and its performance surpassed that of most of the reported ethanolamine sensors. The 2.0 mol% Pd-SnO_2_ gas sensor may thus be seen as a promising candidate for practical ethanolamine detection applications.

## 4. Gas-Sensing Mechanism

SnO_2_ is a typical n-type metal oxide semiconductor material, and its sensing mechanism can usually be described in detail using the space charge model. When pure SnO_2_ and Pd-SnO_2_ samples are exposed to air, oxygen molecules adsorb onto the material surface and form negative oxygen ions by capturing free electrons from the conduction band of the material. Under various temperature conditions, surface chemically adsorbed oxygen anions can exist in different forms. The reaction process can be described in detail as follows [22,43]:

(1) $$O_{2}\left(gas\right)\to O_{2}\left(ads\right)$$

(2) $$O_{2}\left(ads\right)+e^{-}\to O_{2}^{-}\left(ads\right)\;\left(T<100\;℃\right)\;\;$$

(3) $$O_{2}^{-}+e^{-}\to {2O}^{-}\left(ads\right)\;\left(100\;℃<T<300\;℃\right)\;$$

(4) $$O^{-}\left(ads\right)+e^{-}\to O^{2-}\left(ads\right)\;\;\left(T>300\;℃\right)$$

**Figure 5:**
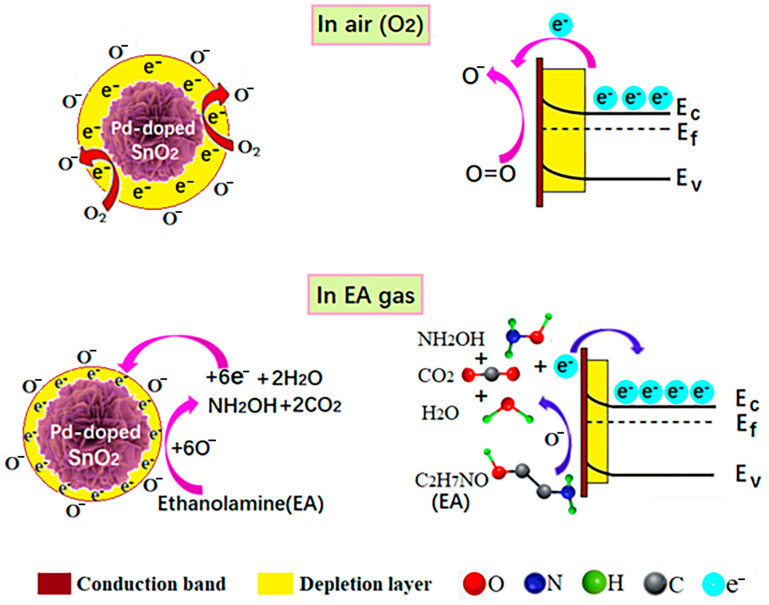
The gas-sensing mechanism of the Pd-SnO_2_ flower-like hierarchical structure.

During this study, we utilized gas-sensing test methods to identify the optimal operating temperature for the material, and this was found to be 150 °C. At this temperature, the adsorbed oxygen species on the material’s surface primarily take the form of O⁻. With the formation of O⁻ ions, the electron concentration on the surface of SnO_2_ decreases, leading to a decline in conductivity. At the same time, the energy band of SnO_2_ undergoes an upward bending, and an electron depletion layer is formed on the surface of the SnO_2_ nanomaterial. Upon exposure to ethanolamine gas, ethanolamine molecules react with oxygen anions adsorbed on the SnO_2_ surface. This reaction returns electrons to the conduction band, increasing the electron concentration and narrowing the electron depletion layer, thereby enhancing conductivity. The reaction process can be described as in Equation (5), and the aforementioned gas-sensing mechanism is illustrated in Figure 5 [44,45].

(5) $$H_{2}NCH_{2}CH_{2}OH+6O^{-}\to NH_{2}OH+2H_{2}O+2CO_{2}+6e^{-}$$

Several reasons may be given for the superior gas-sensing performance of Pd-SnO_2_ gas sensors, compared with pure SnO_2_ gas sensors. According to the results of the XRD analysis, the doping of Pd into SnO_2_ nanoparticles inhibits the growth of SnO_2_ crystal grains, thereby enhancing the gas response. Pd doping introduces lattice defects into SnO_2_, and these serve as active sites for gas adsorption. Analysis of the XPS O1s spectrum revealed that the oxygen vacancy content in 2 mol% Pd-SnO_2_ was 33.27%, higher than the 10.62% in pure SnO_2_. The XPS Pd 3D spectrum confirmed the presence of Pd^2+^ in the sample of Pd-SnO_2_. The formation of PdO on the SnO_2_ surface further attracts electrons from the SnO_2_ conduction band, generating more oxygen anions. The chemical adsorption oxygen content of Pd-SnO_2_ was 18.02%, higher than the 15.92% in pure SnO_2_. This increase in chemical adsorption oxygen content enhances the reaction between Pd-SnO_2_ and target gases, potentially leading to superior sensing performance, compared with pure SnO_2_.

First-principles calculations based on density functional theory (DFT) are extensively employed for in-depth analysis of the mechanisms of gas-sensitive materials. According to the literature, the (110) surface of SnO_2_ exhibits the highest thermal stability [24]. Consequently, this crystal face was chosen as the model for studying gas adsorption. The equilibrium lattice constants of the rutile-type SnO_2_ unit cell were optimized using a 10 × 10 × 16 Monkhorst–Pack k-point grid for Brillouin zone sampling to a = b = 4.806 Å and c = 3.230 Å. This optimized structure was then used to construct a SnO_2_ (110) surface model with p (4 × 2) periodicity in the x and y directions, consisting of two stoichiometric layers in the z direction separated by a vacuum layer 15 Å deep to separate the surface slab from its periodic duplicates. This model contained 48 Sn and 96 O atoms. During structural optimizations, the gamma point in the Brillouin zone was used for k-point sampling, and only the top stoichiometric layer was allowed to relax, while the bottom layer was fixed.

**Figure 6:**
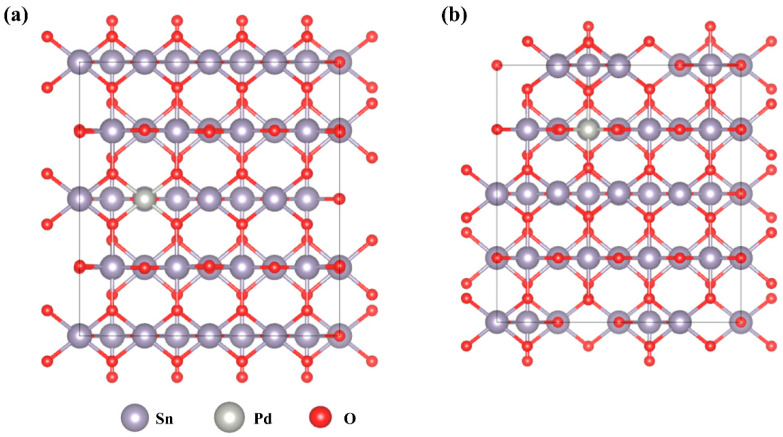
Top views of Pd^2+^ doped SnO (110) surface for (**a**) Sn_5c_, (**b**) Sn_6c_.

Typically, dopant atoms tend to replace Sn atoms in the surface layer of SnO_2_. In the SnO_2_ (110) plane model, there are two types of surface Sn atoms: 5-coordinate (Sn_5c_) and 6-coordinate (Sn_6c_). The optimized model is illustrated in Figure 6.

In the present study, to further investigate the adsorption of ethanol, acetone, formaldehyde, ether, and ethylamine on the three structural models (the optimized SnO_2_ (110) surface, Pd atoms replacing Sn5c atoms, and Pd atoms replacing Sn6c atoms), we calculated the surface adsorption energies of these gas molecules on the three models. The adsorption energy (E_ads_) of adsorbent A, which included ethanol, acetone, formaldehyde, ether, and ethylamine, was calculated using Equation (6) [46].

(6) $$E_{ads}=E_{A/surf}-E_{surf}-E_{A\left(g\right)}$$

In this context, E_A/surf_ signifies the total energy of the system following the adsorption of adsorbent A onto the model surface, whereas E_surf_ refers to the energy of the clean surface. E_A(g)_ denotes the energy of an isolated A molecule within a cubic periodic box with a side length of 10 Å, sampled with a 1 × 1 × 1 Monkhorst–Pack k-point grid for Brillouin zone sampling. Table 3 presents the calculated adsorption energy data for pure SnO_2_ and Pd-doped SnO_2_ surfaces in relation to the five aforementioned gases. The data in the table reveal that ethanolamine exhibited the strongest adsorption capability on both the optimized SnO_2_ (110) and Pd-doped SnO_2_ (110) surfaces, compared with other gases. Specifically, in the structural model where Pd atoms replaced Sn6c atoms, the adsorption energy of ethanolamine molecules was higher than that of the other two models, a result which suggests that this model is more favorable for ethanolamine adsorption. We may say, then, that Pd doping significantly enhances the response and selectivity of SnO_2_-based gas sensors to ethanolamine gas.

**Figure 7:**
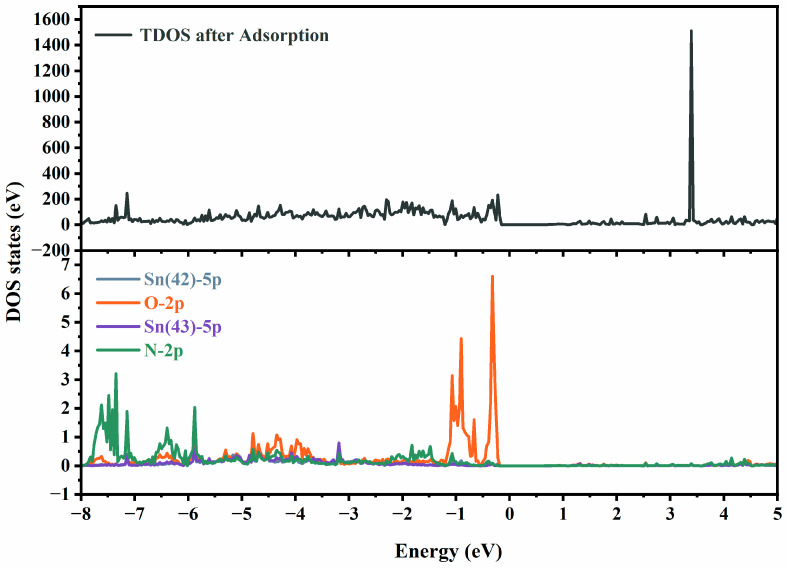
Total density of states and partial density of states of Pd-doped SnO_2_ (110) surfaces after adsorption of ethanolamine.

To thoroughly investigate the influence of ethanolamine adsorption on the electronic structure at the atomic configuration where Pd atoms replace Sn6c atoms, we conducted a detailed analysis of the density of states (DOS) following ethanolamine adsorption, and the results are shown in Figure 7. From the diagram, it is evident that the 5p orbital of Sn (42) forms a bond with the 2p orbital of oxygen, while the 5p orbital of Sn (43) bonds with the 2p orbital of nitrogen. Additionally, the impurity levels near the Fermi level after adsorption are primarily due to the bonding between oxygen and Sn (42) as well as nitrogen and Sn (43). In the aforementioned system, the strong interaction between Sn and O elements, as well as with N, results in the system exhibiting the highest ethanolamine adsorption capacity. This finding aligns with the results of theoretical calculations and further confirms the high sensitivity of the material to ethanolamine gas.

## 5. Experimental Section

### 5.1. Materials

Stannous sulfate (SnSO_4_) and trisodium citrate (C_6_H_5_Na_3_O_7_·2H_2_O) were purchased from Sinopharm Chemical Reagent Co., Ltd. (Shanghai, China). Palladium nitrate (Pd (NO_3_)_2_·2H_2_O) was provided by Macklin Biochemical Co., Ltd. (Shanghai, China). All of the chemical reagents used were of analytical grade and were used directly without further purification.

### 5.2. Preparation of the Pd-SnO_2_ Flower-like Hierarchical Structure

Using a typical synthesis process, 1.08 g of SnSO_4_, 2.95 g of C_6_H_5_Na_3_O_7_·2H_2_O, and a certain amount of Pd (NO_3_)_2_·2H_2_O were dissolved into 60 mL of mixed solution (ethanol/deionized water = 1:2) and vigorously stirred for 60 min. The corresponding molar ratios between the elements Pd and Sn were 0 mol%, 0.5 mol%, 1.0 mol%, 1.5 mol%, 2.0 mol%, 2.5 mol%, and 3.0 mol%. The mixed solution was transferred to a Teflon-lined stainless-steel autoclave (100 mL), and a hydrothermal reaction was performed at 190 °C for 26 h. After natural cooling to room temperature, the precipitate was centrifuged several times with deionized water and anhydrous ethanol. The centrifuged product was then dried at 80 ·C for 12 h. Finally, the centrifuged product was calcined at 500 °C for 2 h in a muffle furnace to obtain the final powder.

### 5.3. Sensor Fabrication and Gas-Sensing Properties Test

The preparation of gas sensors and the gas-sensing properties test were based on previously published reports in the literature [47]. The typical sensor fabrication process was as follows: First, 0.015 g of prepared SnO_2_ sample was placed in an agate mortar, to which an appropriate amount of anhydrous ethanol was added; mixing and grinding were then carried out for about 1 h until a uniform slurry was formed. Next, using a fine-haired brush, this slurry was evenly coated onto the surface of a ceramic tube so that a thin film of sensitive material was formed. When coating was complete, the sensor was placed at room temperature and allowed to dry naturally. A Ni–Cr alloy heating wire was then inserted into the ceramic tube, and the tube was then welded to the base of the sensing element. Finally, the prepared gas sensor was placed on an aging platform and aged at 300 °C for 160 h.

**Figure 8:**
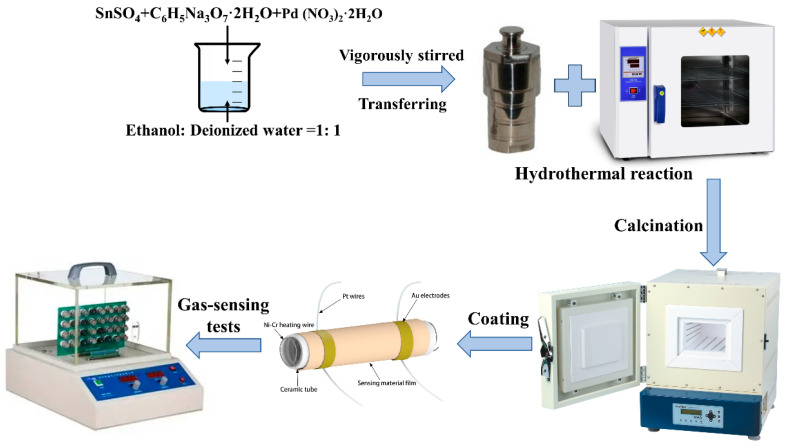
Schematic diagram for the preparation of Pd-SnO_2_ hierarchical structure and gas-sensing tests.

To evaluate the gas-sensing performance of the samples, a series of tests were conducted using the WS-30B (Weisheng Instruments Co., Zhengzhou, China) gas-sensor test system. In the present study, we used a static gas distribution method to test gas-sensing performance. First, the gas sensor was placed within the gas chamber; then, we waited for its resistance in air to stabilize. The resistance value recorded at this time was defined as R_a_. Next, the gas to be tested was injected into the chamber. Once the sensor’s resistance stabilized again in the target gas environment, the resistance value was recorded; this was defined as R_g_, and the sensor response was defined as S = R_a_/R_g_. The response/recovery time was the time required for the resistance value signal of sensitive materials to reach 90% of the total change after contacting the target gas or recovering to the air atmosphere. The preparation processes for the Pd-SnO_2_ hierarchical structure and gas-sensing tests are depicted in Figure 8.

### 5.4. Characterization

The structure and composition of the materials were characterized by means of X-ray powder diffraction (XRD, Bruker D8 Advance, Bruker, Bremen, Germany, with Cu Kα radiation (λ = 0.15406 nm)). XRD data were recorded from 5 to 90° with a scan rate of 5° min^−1^. The microscopic morphology and size of the samples were recorded by a field emission scanning electron microscope (FESEM, Gemini SEM 300, Carl Zeiss, Oberkochen, Germany) equipped with an EDX detector. Energy-dispersive X-ray spectroscopy (EDX, XFlash610-H, Bruker, Berlin, Germany) was used to analyze the elemental distribution of the samples. To obtain more detailed information about the morphology and structure of the samples, observations were made using transmission electron microscopy and high-resolution transmission electron microscopy (HRTEM, JEM-2100, JEOL, Tokyo, Japan, with an acceleration voltage of 200 kV). X-ray photoelectron spectroscopy (XPS, Thermo Scientific, ESCALAB 250XI, Waltham, MA, USA, with Al K_α_ excitation energy of 1486.6 eV) measurements were used to analyze the chemical state of the samples, with charge correction referenced to the standard binding energy of 284.80 eV for the C 1s peak.

### 5.5. DFT Calculation

We employed the Vienna Ab initio Simulation Package (VASP) to perform all density functional theory calculations. These calculations were conducted using the Perdew–Burke–Ernzerhof (PBE) functional under the generalized gradient approximation (GGA) for the correction of the exchange-correlation potential [48,49,50]. During the computations, we used projector augmented-wave (PAW) potentials to represent the ionic cores, and plane-wave cutoff energy was set to 400 eV [51]. Under the Gaussian smearing method, partial occupancies of the Kohn−Sham orbitals are allowed with a width of 0.05 eV [52,53]. We considered the electronic energy to be self-consistent when the energy change was less than 1 × 10^−5^ eV, and geometric optimization was deemed convergent when the force change on each atom was less than 0.02 eV/Å [54]. To describe the dispersion interactions, we utilized the DFT-D3 method of Grimme [55].

## 6. Conclusions

In the present study, we successfully fabricated Pd-doped SnO_2_ flower-like hierarchical structures using a simple one-step hydrothermal method, where the nanosheets had a thickness of approximately 30 nm. The developed sensor exhibited excellent sensitivity to ethanolamine at relatively low operating temperatures, achieving a high detection response value of 106 to 100 ppm ethanolamine at 150 °C. The sensor also demonstrated rapid response and recovery times of 10 s and 12 s, respectively. Furthermore, it exhibited outstanding selectivity and long-term stability. By doping Pd into the SnO_2_ lattice, numerous oxygen vacancy defects were generated on the material’s surface. These defect sites significantly enhanced the gas-sensing reaction, thereby improving the material’s gas sensitivity. DFT calculations revealed that in the optimized adsorption model, there was a strong interaction between Sn, O, and N. In summary, this study not only experimentally verified the potential application of Pd-SnO_2_ flower-like hierarchical structures for detecting ethanolamine gas, but also supported the scientific basis of this discovery through theoretical calculations, opening up new avenues for improving the gas sensitivity of SnO_2_-based materials.

## Figures and Tables

**Table 1:** Structural data and refinement parameters for the SnO_2_ hierarchical structure calculated by Rietveld refinement of the experimental XRD powder pattern.

Space Group	P4_2_/mnm	
Lattice parameters	a (Å)	4.7427
	b (Å)	4.7427
	c (Å)	3.1907
Unit cell volume (Å^3^)	71.7674	
Element coordinates Sn	x	0
	y	0
	z	0
O	x	0.3024
	y	0.3024
	z	0
Average grain size (nm)		9.3810
Average maximum microstrain		0.0022
R_wp_ (%)		8.48%
GOF-index		1.37

**Table 2:** Performance comparison of ethanolamine gas sensors for various metal oxide semiconductors.

Materials	Con. (ppm)	T (°C)	Res.	Ref.
Popcorn-shaped yeast@WO3	100	180	6.80	[3]
Au-ZnO hollow microsphere	100	240	68.8	[4]
Au/ZnO rod-like nanoflower	100	210	138.3	[39]
S-ZnO nanoflower	100	240	84.0	[40]
WO_3_-SnO_2_ nanocomposite	50	220	13.0	[41]
Ce-doped ZnO thin films	100	25	65.0	[42]
Pd-SnO_2_ nanoflower	100	150	106	This work

**Table 3:** The calculated adsorption energy (eV) from gas molecules onto pure SnO_2_ and Pd-doped SnO_2_.

Adsorption Models	Adsorbents	E_A(g)_ (eV)	E_surf_ (eV)	E_A/surf_ (eV)	E_ads_ (eV)
Optimized SnO_2_ (110) surface	ethanol	−46.940	−580.882	−629.756	−1.934
	acetone	−56.041	−580.882	−638.256	−1.333
	formaldehyde	−22.163	−580.882	−604.159	−1.114
	ether	−79.841	−580.882	−661.198	−0.475
	ethylamine	−58.630	−580.882	−641.902	−2.390
Pd atoms replacing Sn_5c_ atoms	ethanol	−46.940	−886.404	−934.035	−0.691
	acetone	−56.041	−886.404	−944.191	−1.746
	formaldehyde	−22.163	−886.404	−910.178	−1.610
	ether	−79.841	−886.404	−966.752	−0.507
	ethylamine	−58.630	−886.404	−948.017	−2.983
Pd atoms replacing Sn_6c_ atoms	ethanol	−46.940	−886.714	−935.727	−2.073
	acetone	−56.041	−886.714	−944.239	−1.484
	formaldehyde	−22.163	−886.714	−910.938	−2.061
	ether	−79.841	−886.714	−967.050	−0.495
	ethylamine	−58.630	−886.714	−949.151	−3.807

## Data Availability

The data can be provided on the responsible request to the corresponding author.
